# Implementing active surveillance for TB: A descriptive survey of healthcare workers in the Eastern Cape, South Africa

**DOI:** 10.4102/phcfm.v16i1.4217

**Published:** 2024-02-23

**Authors:** Febisola I. Ajudua, Robert J. Mash

**Affiliations:** 1 Department of Family Medicine and Primary Care, Faculty of Medicine and Health Sciences, Stellenbosch University, Cape Town, South Africa; 2 Department of Family Medicine and Rural Health, Faculty of Health Sciences, Walter Sisulu University, Gqeberha, South Africa; 3 Faculty of Health Sciences, Nelson Mandela University, Gqeberha, South Africa

**Keywords:** community-orientated primary care, primary health care, tuberculosis, disease surveillance, community health worker

## Abstract

**Background:**

South Africa is a tuberculosis (TB) high-burden country. In the Eastern Cape (EC), community health worker (CHW) teams implement active surveillance for TB to curb spread in disadvantaged communities. However, achieving the goals of the End-TB strategy require coordinated efforts that implement policy and strengthen health systems.

**Aim:**

This survey described views of healthcare workers (HCWs) in primary care facilities on factors that influence implementation of active surveillance for TB.

**Setting:**

This survey was conducted across two districts, among healthcare workers working in TB rooms at primary health facilities.

**Method:**

A cross-sectional survey of HCW in the EC.

**Results:**

The survey included 37 clinics in the OR Tambo Health District (ORTHD) and 44 clinics in the Nelson Mandela Bay Health District (NMBHD). Routine screening at primary care facilities (88.2%) and contact tracing initiatives (80.8%) were the common modes of TB screening. Tuberculosis screening services in the community were only provided by CHWs in 67.3% of instances. Although CHWs were adequately trained and motivated; the lack of transport, limited availability of outreach team leaders (OTLs) and poor security limited implementation of TB screening services in the community. Comparison between both districts revealed TB screening was limited by lack of transport in the rural district and poor security in the urban context. Community engagement provided a platform for improving acceptability.

**Conclusion:**

Community-based TB screening was limited. Inadequate coordination of services between stakeholders in the community has limited reach. Further research should describe that coordinating resource allocation and community empowerment could improve the implementation of active surveillance for TB.

**Contribution:**

This study highlights the views of TB room HCWs who believe the opportunity for community-level TB screening is improved with effective leadership and community engagement for acceptability of these services.

## Introduction

In 2020, there were thought to be 10 million people worldwide with active tuberculosis (TB); however, only 5.8 million cases were reported.^1^ This gap has widened as a direct impact of the COVID-19 pandemic on global health systems.^1^ It is evident that achieving the goals of the End-TB strategy will require an extra effort to implement policy and win back the ground loss on TB control. It is also evident that creating healthy and resilient communities is a necessity for achieving the sustainable development goals (SDGs).^2^ The worldwide effort to curb the COVID-19 pandemic shows that much can be achieved by cohesive action between politicians and leaders of health systems.^3^ Tuberculosis affects mainly low- and middle-income countries and is especially rampant among vulnerable populations in these countries.^4^ In order to achieve the End-TB goals, active surveillance for TB with linkage to care that leads to a reduction in transmission is essential.^5^ Active surveillance for TB can be expensive when implemented as a standalone intervention, but it remains the best option for early detection in settings with a high burden of TB.^6,7,8^ Providers of TB services should implement strategies that are feasible and sustainable, considering the context and resources available.^6,9^

In South Africa, TB incidence is estimated at 554/100 000.^10^ Despite the successes of the South African national tuberculosis programme (NTP) in the last two decades, South Africa still has a high burden of TB. The estimated number of active TB cases in 2020 was 328 000; however, only 191 074 were notified. This implies that a significant number of cases were undiagnosed or not reported. It can be inferred that the missing cases were mainly due to people not accessing health services. The health system in South Africa was badly affected in 2020 by COVID-19 and resources were diverted to address the pandemic.^11^ This meant that services for TB were less available, although in some contexts innovative ideas were implemented to assist patients in accessing services.^12^ Patients with TB and HIV could not access health services for diagnosis or continued therapy. This revealed the need for a resilient South African health system that can continue functioning despite new public health challenges.^13,14^

The community-based services provided by community health worker (CHW) teams, also called ward-based primary health care outreach teams (WBPHCOTs), provide screening services for a number of diseases.^15^ Active surveillance for TB is one of the services provided by these teams.^16^ A number of initiatives are reported that show the contribution of CHW teams to the response to the COVID-19 pandemic.^17^ They are known to improve coverage of the population and access to the health system.^18,19^ It is understood that the full impact of the COVID-19 pandemic on the TB epidemic in high-burden countries is only becoming visible as the pandemic subsides.^1^ The impact reported by the South African NTP includes an increase in the number of people with active TB, many of whom have inadequate access to services, and an increase in the number of patients with undiagnosed TB.^20^ It is essential that active surveillance for TB by CHW teams is maintained to support early diagnosis and linkage to care. Research work from countries with a high burden of TB reveals that active surveillance for TB in communities, especially among vulnerable populations, has a high yield in identifying patients with active TB.^21,22^

Active surveillance for TB is currently identified as the best strategy for early diagnosis of TB to curb the spread in communities.^5,17^ It is conducted in South Africa as symptom screening initiated outside of health facilities in at-risk populations. This is seen in workplaces, prisons, schools and households, which are at increased risk due to socioeconomic factors that drive the transmission of TB. The NTP also recommends intensified case-finding initiatives within health facilities. In these instances, patients visit health facilities for other reasons, but are screened for symptoms of TB. These are also recognised TB screening initiatives, but cannot be considered active surveillance as the encounter is initiated by the people seeking healthcare. The literature often uses active surveillance and active case finding interchangeably.^23^

In South Africa, the CHW teams are known to assist with active surveillance in the community.^24^ Some of the methods used include contact tracing of known cases, campaigns in the community and symptom screening during household visits. The implementation of these strategies is well described as part of community-based services with experienced CHW teams in a number of settings.^25,26^ However, it is understood that though active surveillance is included in the scope of work for CHW teams, implementation varies between locations and is influenced by a number of factors that are not always directly linked to the health system.^27^

An important factor is the relationship between the CHW teams and other stakeholders in the community, including community leaders.^16^ These relationships have the capacity to influence the effect of the CHWs on the social determinants of health in the community.^28^ Active surveillance by CHWs is part of a complex network of factors that influence how quickly the client presents to the facility.^16,29^ Some of these include cost of transport, geographical access, level of education, perceived quality of services in the facility and belief systems.^30^ A number of factors directly influence the outcomes of active surveillance for TB.^29^ These include the availability of resources for CHWs such as stationery, transport, training and support from the facility.^31^

The Eastern Cape is home to over 6 million South Africans with high levels of poverty, unemployment and poor housing.^32^ It is ranked among the top three provinces with the highest incidence of TB in South Africa at 692/100 000 population.^1^ Tuberculosis is still a leading cause of mortality, and one of the contributing factors is delayed diagnosis.^33^ Previous qualitative studies identified factors that influence the successful implementation of active surveillance for TB.^34^ The factors were categorised into factors related to the CHW teams, factors related to patients, factors related to the broader health services and factors related to the community. However, these factors have not been quantified in the Eastern Cape. This study aimed to describe the views of healthcare workers from TB services at primary care facilities on the factors that influence the implementation of active surveillance for TB across a rural and an urban district in the Eastern Cape.

## Research methods and design

### Study design

This was a cross-sectional survey of healthcare workers on factors influencing the implementation of active surveillance for TB in the Eastern Cape province of South Africa.

### Setting

The Eastern Cape province consists of six rural districts and two metropolitan areas. Professional nurses provide TB diagnosis and treatment services at primary care facilities across the province. These facilities provide services to surrounding communities that are divided into municipal wards.^15^ Often, a facility is affiliated to only one WBPHCOT. The WBPHCOT provides community-based services to the surrounding wards. Each CHW should be responsible for 250 households, but often works over a larger area due to the shortage of CHWs. The team linked to a facility often work in all the municipal wards linked to that facility, although the original intention was one team per ward.

Community health worker teams are meant to provide active surveillance for TB in the community. They work in WBPHCOTs together with the TB room nurses in contact tracing for identified cases and provide TB screening services during home visits and community campaigns. These teams should consist of six CHWs and an outreach team leader (OTL). The OTL is often a professional nurse skilled in primary health care. The CHWs are trained prior to starting work in a number of activities, including screening for TB. The TB screening service involves symptom screening of clients in high-risk population groups. Several nongovernmental organisations (NGOs) also provide services in communities across the province via CHWs. These CHWs work independently of the WBPHCOTs, who are employed by the Department of Health (DoH). The NGOs may provide TB-related services or focus on other health priorities.

### Selection of clinics and respondents

**FIGURE 1:**
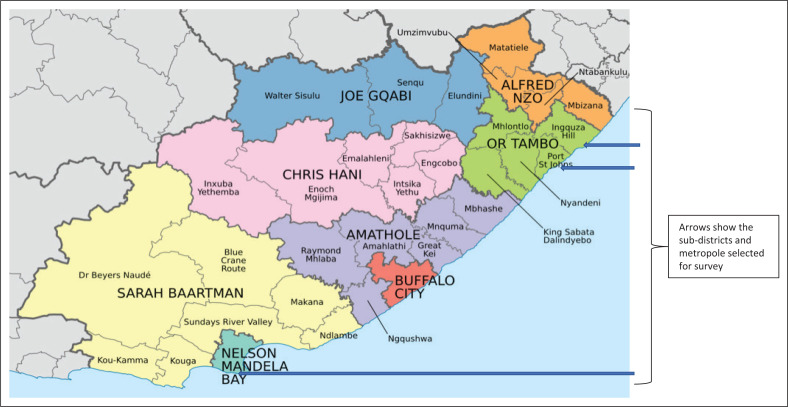
Map of the Eastern Cape showing the districts and subdistricts.

The two most populous rural and urban districts were purposively selected. The Nelson Mandela Bay Health District (NMBHD) is an urban district (see Figure 1) made up of three subdistricts. Forty-four primary care facilities (clinics and community health centres) were identified across the district. The OR Tambo Health District (ORTHD) is mainly rural (see Figure 1) and contains five subdistricts. Two rural subdistricts were randomly selected, after excluding the peri-urban district. These were Port St John’s and Inguza Hill subdistricts with 37 clinics identified across both subdistricts. All primary care facilities in NMBHD and the selected subdistricts in ORTHD were included in the survey. At these primary care facilities, the nurse in charge of the TB service was selected.

### Development of the questionnaire

The questionnaire was developed after review of the literature and two previous qualitative studies that explored the key factors related to implementation of active surveillance for TB.^34,35^ The draft questionnaire was content validated by a panel of five experts. Panel members were asked to consider if all relevant issues had been included, if any irrelevant issues were included and to give feedback on the construction of questions for these issues. The panel consisted of experts in research and the NTP. They gave feedback over two iterations of the instrument.

The final questionnaire included questions on demographics and the methods of providing TB surveillance in the facilities and surrounding communities. It also included statements on factors known to influence TB screening services and required respondents to indicate their level of agreement with these statements. Respondents were also allowed to provide further comments in each category based on their experience. This afforded an opportunity for feedback on factors that may have been unique to the context of each respondent.

The questionnaire was piloted with two healthcare workers (HCWs) in the TB services at two facilities in the Sarah Baartman Health District of the Eastern Cape. Feedback from the nurses revealed that questions were clear and there was no need for revision.

### Data collection

The researcher contacted the nurses telephonically to introduce the survey and explained the process of electronic data collection using REDCap software. The survey link was provided to all potential participants via email. Data were collected between May and August 2021. Nurses received a series of emails and telephonic reminders to complete the survey during this period.

### Data analysis

Data were exported from REDCap and analysed by the first author using the Statistical Package for Social Sciences (SPSS) software version 27. The data were categorical and described by means of frequencies and percentages. Any differences in the responses between the urban and rural districts were tested for, using the chi-square test.

### Ethical considerations

Ethical approval was obtained from the Health Research Ethics Committee (HREC) of Stellenbosch University, reference S17/10/189_1243. Permission to conduct the survey was received from the Eastern Cape Department of Health EC_201801_013. Each person was at liberty to consent or refuse participation. Ethical approval was granted from the human research ethics committee. The National health research database, requires the researcher to obtain the consent of the head of institutions before conducting research in their facilities. The permission is required in addition to the ethics approval to move ahead with the study.

## Results

The survey included 23 rural participants from the ORTHD and 30 urban participants from the NMBHD. The response rate was 64.8% in the ORTHD and 63.6% in the NMBHD. Each participant was representative of one clinic.

### Demographic data

Most respondents were professional nurses (Table 1) and 51 (96.2%) were women. Their work experience ranged from 1 to 39 years, with a mean of 15 years (s.d. = 11). A comparison of demographic characteristics revealed no significant differences between the districts.

**TABLE 1:** Demographic characteristics of the respondents.

Variable	Total		Urban		Rural		*P*
	*n*	%	*n*	%	*n*	%	
**Age (years) (*N* = 52)**							0.890
20–29	10	19.2	5	17.2	5	21.70	
30–39	8	15.4	4	13.8	4	17.44	
40–49	14	26.9	7	24.1	7	30.40	
50–59	17	32.7	11	37.9	6	26.10	
> 60	3	5.8	2	6.9	1	4.30	
**Occupation (*N* = 52)**							0.269
Professional nurse	50	96.2	29	100.0	21	91.30	
Enrolled nurse	1	1.9	0	0.0	1	4.30	
CHW	1	1.9	0	0.0	1	4.30	
Other	0	0.0	0	0.0	0	0.00	
**Work experience (years) (*N* = 53)**							0.614
0–4	11	20.8	5	16.7	6	26.10	
5–9	11	20.8	4	13.3	7	30.40	
10–14	10	18.9	6	20.0	4	17.40	
15–19	3	5.7	2	6.7	1	4.30	
20–24	4	7.5	3	10.0	1	4.30	
25–29	6	11.3	4	13.3	2	8.70	
> 30	8	15.1	6	20.0	2	8.70	
**Work experience in TB (years) (*N* = 53)**							0.099
0–4	27	50.9	14	46.7	13	56.50	
5–9	16	30.2	8	26.7	8	34.80	
10–14	4	9.4	5	16.7	0	0.00	
15–19	1	1.9	0	0.0	1	4.30	
20–24	3	5.7	3	10.0	0	0.00	
25–29	1	2.0	0	0.0	0	0.00	
> 30	1	1.9	0	0.0	1	4.30	

TB, tuberculosis.

Table 2 shows the methods of identifying patients with active TB across both districts. The commonest forms of TB screening were symptom screening in the facilities (88.5%) and contact tracing by CHWs (80.8%). Only 67.3% of clinics had household screening by CHWs. More than half of the clinics also conducted contact tracing by telephone (57.7%).

**TABLE 2:** The availability of different methods of tuberculosis screening (*N* = 52).

Method	Frequency	%
Screening at primary care facilities	46	88.5
Contact tracing by community health workers	42	80.8
Household screening by community health workers	35	67.3
Telephonic contact tracing by TB room staff	31	57.7
Occasional door to door campaigns	25	48.1
Screening in schools	21	40.4
Screening at mobile clinics	15	28.9
Screening at public gatherings	10	23.1
Screening by private general practices	6	11.5

TB, tuberculosis.

Table 3 summarises those providing community-based TB screening services. The commonest providers of TB screening were WBPHCOTs (69.8%) and CHWs funded by NGOs (54.7%). In 35.8% of clinic catchment areas, both role players were providing similar services, while in 5.7% of clinics no one was providing services. Overall, 88.7% of clinics had CHWs to provide TB screening services in the community.

**TABLE 3:** Community-based tuberculosis screening service providers in communities (*N* = 53).

Service provider	Frequency	%
Ward-based PHC outreach teams only	18	34.0
Nongovernment organisations only	10	18.9
Both available	19	35.8
Professional nurse	1	1.9
None	3	5.7
Don’t know	2	3.8

PHC, primary health care.

### Factors related to community health workers and community-based tuberculosis screening in communities

The majority of respondents agreed that the CHWs received standardised and adequate training for community-based TB screening services (Table 4). A majority (80.8%) also agreed CHWs could not visit all the households in their specified wards because of a lack of transport. Most respondents reported that CHWs had identification tags and uniforms (66.7%) as well as stationery (76.9%). Although 76.9% of respondents agreed that CHWs were motivated, 40.3% said that they did not have enough time for TB screening services.

**TABLE 4:** Factors related to community health workers and community-based tuberculosis screening.

Variables	Level of agreement							
	Strongly agree		Agree		Disagree		Strongly disagree	
	*n*	%	*n*	%	*n*	%	*n*	%
There is a standardised training course on TB screening for community health workers across my district (*N* = 50)	6	12.0	29	58.0	13	26.0	2	4.0
Community health workers are adequately trained to provide TB screening (*N* = 52)	12	23.1	27	51.9	10	19.2	3	5.8
Community health workers are provided with uniforms and name tags for identification (*N* = 51)	10	19.6	24	47.1	14	27.5	3	5.8
Community health workers have stationery for household assessments and referrals (*N* = 52)	10	19.2	30	57.7	8	15.4	4	7.7
Community health workers cannot visit all their households because of a lack of transport (*N* = 52)	17	32.7	25	48.1	9	17.3	1	1.9
Community health workers are motivated to perform TB screening services (*N* = 52)	9	17.3	31	59.6	10	19.2	2	3.9
Community health workers have enough time to provide TB screening services (*N* = 52)	7	13.5	24	46.2	21	40.3	0	0.0
Differences in the conditions of employment for community health workers between nongovernmental organisations and Department of Health are a challenge to TB services (*N* = 51)	12	23.5	25	49.1	12	23.5	2	3.9
Community health workers educate the community about the symptoms of TB (*N* = 52)	19	36.5	30	57.7	2	3.9	1	1.9
Facility-based and community-based TB services coordinate their TB services activities (*N* = 52)	16	30.8	27	51.9	8	15.4	1	1.9
All community members served by this facility have access to services from community health workers (*N* = 52)	15	28.8	31	59.6	3	5.8	3	5.8

TB, tuberculosis.

Other factors mentioned by respondents were a shortage of CHWs, use of CHWs to provide services in the facility and not the community, poor coordination between the facility and community-based CHW teams, the impact of COVID-19 on TB services, and the difficulty of screening community members who were employed.

### Factors related to patients

Most respondents (78.8%) agreed with the statement that patients were in the habit of attending different primary care facilities (Table 5). The majority thought that patients did not provide correct home addresses (69.2%) or cell phone information (65.0%). Also, 75.0% of respondents agreed that patients who were disadvantaged socioeconomically presented with more advanced TB symptoms. In response to the open question, several respondents stated that CHWs still faced challenges with the acceptability of their services, and many experienced aggression or verbal abuse in the community from people reluctant to open their homes to them.

**TABLE 5:** Factors related to patients with regard to community-based tuberculosis screening services.

Variables	Levels of agreement							
	Strongly agree		Agree		Disagree		Strongly disagree	
	*n*	%	*n*	%	*n*	%	*n*	%
Patients choose to attend different primary care facilities for care (*N* = 52)	12	23.0	29	55.8	8	15.4	3	5.8
Patients provide accurate home addresses (*N* = 52)	3	5.8	13	25.0	24	46.1	12	23.1
It is difficult for patients to speak privately to the community health worker (*N* = 52)	3	5.8	20	38.5	23	44.2	6	11.5
Patients with fewer social and financial resources often present with more advanced TB symptoms (*N* = 52)	16	30.8	23	44.2	10	19.2	3	5.8
Patients provide reliable contact details, e.g. cell phone number (*N* = 52)	4	7.7	14	26.9	23	44.2	11	21.2

TB, tuberculosis.

### Factors related to the facility and district services

Table 6 presents factors related to the broader health services. Almost all respondents (88.2%) agreed that the nurse in charge of the TB room was adequately trained, although recruiting staff for TB services was difficult (69.2%). Seventy-five percent of respondents agreed that the lack of OTLs made it difficult to supervise TB screening activities in the community. According to 70.6% of respondents, there was a reliable record of patients referred by the CHW teams with TB symptoms. Also, 85.0% agreed that their facility had reliable and routinely available information on the community-based TB screening services in the community. A sizeable proportion (42.3%) of respondents thought that there were problems with collaboration between health and social services. In other comments, some respondents added that there was inadequate support for the community-based TB screening services from the facility-based services. Some emphasised the poor coordination of referrals from employers and people outside of the public health system to facilities, delaying the time to definitive diagnosis. Also, a number mentioned poor geographic access to facilities in the rural areas.

**TABLE 6:** Factors related to the facility and district services with regard to community-based tuberculosis screening.

Variables	Level of agreement							
	Strongly agree		Agree		Disagree		Strongly disagree	
	*n*	%	*n*	%	*n*	%	*n*	%
The nurse in charge of the TB room is adequately trained in TB (*N* = 51)	17	33.3	28	54.9	5	9.8	1	2.0
There is a specific person responsible for TB services in this facility (*N* = 52)	24	46.1	20	38.5	7	13.5	1	1.9
It is difficult to recruit health professionals for TB services (*N* = 52)	11	21.1	25	48.1	14	26.9	2	3.9
There is a reliable record of all patients referred by the community health workers with TB symptoms at the facility (*N* = 51)	11	21.6	25	49.0	12	23.5	3	5.9
The facility has reliable and routinely available information on community-based TB screening (*N* = 52)	12	23.1	27	51.9	10	19.2	3	5.8
The lack of an outreach team leader makes it difficult to supervise the TB screening services (*N* = 52)	9	17.3	23	44.2	16	30.8	4	7.7
There is a reliable record of all parts of the TB contact tracing system (*N* = 51)	11	21.6	23	45.1	17	33.3	0	0.0
The nurse in the TB room provides feedback to the CHWs on their referrals to the facility (*N* = 52)	13	25.0	32	61.5	6	11.5	1	1.9
This district has clear goals for TB screening services in line with the 90:90:90 strategy (*N* = 52)	15	28.8	35	67.3	0	0.0	2	3.9
The health promoter in this facility collaborates with the ward-based primary health care outreach teams	5	9.6	15	28.8	20	38.5	12	23.1
Active surveillance for TB should be limited to certain high-risk groups (*N* = 50)	5	10.0	12	24.0	25	50.0	8	16.0
The TB screening services are effective in identifying people with TB (*N* = 51)	24	47.1	27	52.9	0	0.0	0	0.0
There is good collaboration between health and social services (*N* = 52)	3	5.8	27	51.9	19	36.5	3	5.8
There is an effective forum for engagement of the health facility with the community (*N* = 51)	4	7.8	26	51.0	16	31.4	5	9.8
Some areas in this district are difficult to reach because of poor or no roads (*N* = 51)	17	33.3	23	45.1	6	11.8	5	9.8
The community understands the importance of active TB surveillance (*N* = 51)	8	15.7	31	60.8	12	23.5	0	0.0
TB patients are stigmatised in this community (*N* = 51)	1	2.0	12	23.5	34	66.7	4	7.8

TB, tuberculosis.

### Factors related to the community

Table 7 provides responses to the statements on factors related to the community and TB screening. The majority agreed that community members understood the roles of the CHWs (84.3%). This was supported by the 76.5% who agreed that community members accepted and appreciated the work of the CHWs. Further, the community members (74.0%) and community leaders (76.4%) supported health campaigns. However, only 56.9% agreed that CHWs were safe visiting households.

**TABLE 7:** Factors related to the community and tuberculosis screening services.

Variables	Strongly agree		Agree		Disagree		Strongly disagree	
	*n*	%	*n*	%	*n*	%	*n*	%
Community members understand the roles of the CHWs (*N* = 51)	9	17.6	34	66.7	6	11.8	2	3.9
Community meetings allow opportunities to engage residents on TB matters (*N* = 50)	5	10.0	23	46.0	20	40.0	2	4.0
Health campaigns in the community are supported by community leaders (*N* = 50)	7	14.0	30	60.0	12	24.0	1	2.0
Health campaigns in the community are supported by the community residents (*N* = 51)	4	7.8	35	68.6	11	21.6	1	2.0
CHWs can safely visit all households in the community (*N* = 51)	6	11.8	23	45.1	17	33.3	5	9.8
CHWs are accepted and appreciated in this community (*N* = 51)	8	15.7	31	60.8	10	19.6	2	3.9

CHW, community health worker; TB, tuberculosis.

### Comparing the urban and rural contexts for implementing active surveillance for tuberculosis

Table 8 compares the responses from urban and rural clinics. There were no significant differences in factors related to the CHW teams. In terms of factors related to the patients, the rural patients were seen to be more inclined to provide accurate addresses (*p* < 0.001) and contact details (*p* = 0.004). Respondents had low levels of agreement that people found it difficult to speak privately with their CHW in both contexts; however, this challenge had significantly more agreement in the urban context setting (*p* = 0.034). In terms of factors related to the health services, the TB nurses were perceived to be better trained in the urban setting (*p* = 0.017). There was also more specific leadership and accountability for the TB service in the urban setting (*p* = 0.004). In terms of the community, rural areas had poor road networks which impeded TB screening (*p* = 0.031), while urban areas were perceived to be more dangerous (*p* = 0.031).

**TABLE 8:** Comparison of variables between urban and rural districts.

Variables	Urban district (*N* = 29) (%)	Rural district (*N* = 23) (%)	*P*
**Statements on factors related to CHWs and community-based TB screening**			
Standard training course on TB screening for CHWs	63.0	78.3	0.958
CHWs are adequately trained to provide TB screening	75.9	73.9	0.709
CHWs are provided with uniforms and name tags	65.6	68.2	0.295
CHWs have stationery for household registration	79.3	73.9	0.623
CHWs cannot visit all households because of a lack of transport	75.9	87.0	0.700
CHWs are motivated to perform TB screening	75.9	78.3	0.521
CHWs have enough time to perform TB screening	58.6	60.9	0.609
Differences in conditions of employment for CHWs between the DoH and NGOs are a challenge to TB services	79.3	63.6	0.214
CHWs educate the community about the symptoms of TB	96.6	91.3	0.244
Facility-based and community-based TB services coordinate their activities	72.4	91.3	0.498
All community members served by this facility have access to services by CHWs	93.1	82.6	0.251
**Statements on factors related to the patient**			
Patients choose to attend different primary care facilities	72.4	82.6	0.321
Patients provide accurate home addresses	6.9	60.9	< 0.001
It is difficult for patients to speak privately with CHWs	48.3	39.1	0.034
Patients with fewer social and financial resources often present with more advanced TB symptoms	62.1	91.3	0.094
Patients provide reliable contact details e.g. cell phone.	17.2	56.5	0.004
**Statements on factors related to the facility**			
The nurse in charge of the TB room is adequately trained in TB	96.5	73.9	0.017
There is a specific person responsible for TB services in this facility	96.6	69.6	0.004
It is difficult to recruit health professionals for TB services	72.4	65.2	0.278
There is a reliable record of all patients referred by the CHWs with TB symptoms at the facility	71.4	69.6	0.344
The facility has reliable and routinely available information on the community-based TB	79.3	69.6	0.374
The lack of an outreach team leader makes it difficult to supervise the TB screening services	62.1	60.9	0.844
There is a reliable record of all parts of the TB contact tracing system	64.3	26.1	0.255
The nurse in the TB room provides feedback to the CHWs on their referrals to the facility	89.7	82.6	0.675
The district has clear goals for TB screening services in line with the 90:90:90 strategy	93.1	100	0.437
The health promoter in this facility collaborates with the WBPHCOT	37.9	39.1	0.821
Active surveillance for TB should be limited to certain high-risk groups	32.1	36.4	0.253
The TB screening services are effective in identifying people with TB	100.0	100.0	0.304
There is good collaboration between health and social services	58.6	56.5	0.429
**Statements on factors related to the community**			
There is an effective forum for engagement of the health facility with the community	60.7	56.5	0.832
Some areas in this district are difficult to reach because of the poor or no road network	64.3	95.7	0.031
The community understands the importance of active surveillance for TB	71.4	82.6	0.643
TB patients are stigmatised in this community	32.1	17.4	0.393
Community members understand the role of CHWs	78.6	91.3	0.511
Community meetings allow opportunities to engage residents on TB matters	50.0	63.6	0.502
Health campaigns in the community are supported by community leaders	63.0	87.0	0.223
Health campaigns in the community are supported by community members	67.9	87.0	0.417
CHWs can safely visit all households in the community	42.9	73.9	0.031
CHWs are accepted and appreciated	67.9	87.0	0.366

CHW, community health worker; TB, tuberculosis; NGO, nongovernmental organisations; WBPHCOT, ward-based primary health care outreach teams.

## Discussion

### Summary of key findings

Screening in facilities was the commonest form of identifying people with TB, which is not a form of active surveillance. Contact tracing in the community was the commonest form of more proactive surveillance, but was restricted to the known cases of TB. Only two-thirds of the clinics were engaged with proactive surveillance of the entire population at risk, despite 88.7% having CHWs in the catchment area. It appeared that the available resources were not efficiently used, as 35.8% of clinics had services by both WBPHCOTs and NGOs, while 5.7% had no services at all.

Community health workers in WBPHCOTs were adequately trained and had sufficient resources to perform TB surveillance, although their capacity was limited by the many demands on their time. Responding to the COVID-19 pandemic reduced their capacity for active TB surveillance and some CHWs were pulled into facility-based services. Availability of transport was a problem, as well as poor road networks in rural communities. Community health workers were accepted by community members and their leaders, providing support for their activities. However, security was a problem, especially in the urban context. The lack of OTLs for the teams made supervision difficult, although nurses reported that they had good data on TB surveillance. Collaboration between social and health services was an issue in many areas. Nurses perceived that patients had low affiliation with a specific primary care facility.

### Community-based tuberculosis screening

The Eastern Cape certainly fits the WHO’s description of a context with a high burden of TB where social factors continue to drive transmission of TB and where active surveillance is needed.^7^ The findings suggest a third of clinics did not have active surveillance of the population. It is unclear the extent to which NGOs mitigated this problem as they often provided targeted TB services that may not include TB screening of the population.

The results suggest that active TB surveillance for the population at risk was not a priority and that the model of care was dominated by a facility-based perspective. This is seen in the dominance of facility-based screening and contact tracing. The primary health care team needs to embrace a community-orientated perspective if they are to prioritise proactive population outreach and fully empower WBPHCOTs.^36^ Even the use of the label ‘outreach’ in the name of the team implies that they are extending the services and agenda of the facility, rather than enabling a different community-orientated approach to population health management.^37^ The results may imply that the paradigm shift from a facility-orientated to a community-orientated approach to primary health care has not yet occurred.^38^

Research affirms routine, active case finding in high-risk communities improves TB epidemiology. This is further strengthened with the provision of sputum collection for TB testing services.^39^ The WHO’s model of primary health care recommends integrated health services that bring together primary care and a public health approach, which is another way of formulating such a paradigm shift.^40^ Even when a community oriented primary care (COPC) approach is promoted in policy, local managers may interpret and implement policy through the filter of their own understanding, opinions and personal choices.^41,42^

Screening in schools appeared to be insufficient, especially in the adolescent age groups that present with symptoms very similar to adults.^43^ The school health programme also provides opportunities for health education on TB symptoms, as these children can potentially carry information back into households that may not be accessible to CHWs.

The screening of patients in private GP practices was very low and may be a missed opportunity. There is a substantial group of people who make out-of-pocket payments and come from the more vulnerable parts of the community, especially in GP practices situated in these socioeconomically disadvantaged communities. Such screening, however, as in public sector facilities, is still only targeting those who attend health services and not the whole population.

### Community health workers

The COM-B model suggests that behaviour is linked to the capability, opportunity and motivation to perform the behaviour.^44^ This study suggests that CHWs were capable and motivated, but lacked the opportunity to perform TB surveillance. Community health worker teams need to have a dedicated OTL, who is also equipped to provide supportive supervision.^41,45^ The optimism of nurses with regard to the quality of health information derived from CHWs may need to be evaluated further, as literature suggests this is often a problem, especially in the absence of an effective OTL.^46^ This study suggested that transport in general and topography in rural areas were major impediments to screening of the population, while other resources were mostly present. Providing the CHW teams with all the resources they need has been identified as a key factor elsewhere.^15^ Concerns about security and opportunistic crime have also been reported in the literature and limit the reach of CHWs.^46^ Other factors alluded to include the impact of COVID-19 on CHW activities and pulling them into facility-based services.^43^

In some settings outside of South Africa, experienced CHWs are used for supervision of younger, inexperienced CHWs. This may lower the cost of supervision in the teams, improve the prospects for CHWs as healthcare providers and solve the perennial challenge of finding OTLs for the WBPHCOTs.^47^ In the South African setting, a hybrid could be considered in circumstances without enough CHWs depending on the activities required in the community.

### Patients

Community health workers should systematically visit households in their allocated area. This does not require an accurate address or contact details. They are then able to rely on these household records to find patients during contact tracing. Studies in similar contexts show the improved yield when contact tracing is implemented appropriately.^48^

Patients in socioeconomically disadvantaged circumstances present with more advanced TB due to various factors that limit access to care.^49^ Studies show these patients move address frequently to pursue sources of income or because of rent they cannot afford. Community health workers may need access to resources such as transport and social services if they are to link these patients to care.^50^ Unfortunately, access to these resources was perceived as problematic. Resources should be targeted at those most vulnerable and disadvantaged in the communities served.

### Facility level support

Effective leadership is important for adaptive capacity, coordinating the use of available resources and stakeholders, to address the local health challenges.^51^ This study, like many others, recognised the need for good coordination of services in community-based TB screening to target the areas of greatest need and improve the yield of identified active TB cases in the community.^52,53,54^ A well-designed referral system between the team and the facility facilitates linkage to care. Similarly, the success of TB screening in the community health system is facilitated by the OTL’s availability to the team, to provide leadership, mentor and train the CHWs.^55^ The OTL is strengthened by support from the facility staff who avail the team of support that strengthens their capacity to provide active surveillance for TB. Early diagnosis is only the first step in the patient care cascade to reducing the burden of TB in the community.^42,56^ In this survey, the additional comments of several respondents affirmed TB took a back seat in the health services response to the COVID-19 pandemic, and this was observed in other parts of the South African health system.^12^

### Communities

Acceptability of services provided by the CHW teams is recognised to impact on the expected outcome of community-based TB screening in the community.^57^ Respondents asserted that community meetings did not always provide opportunity for community engagement, a recognised strategy for empowering communities to take responsibility for their own improved health outcomes.^58^

Ultimately, the community should have a say in its own health outcomes. The system currently is designed to provide a service without much contribution from the community, short of providing CHWs. The empowered community with a heightened level of awareness of TB disease would be more responsive in their health-seeking behaviour through peer education.^59^ Security concerns within a community are best managed by educating the community about the services provided by the CHWs and their responsibility to protect these individuals. The concept of community health systems is still developing and there is room for further research in the implementation of active surveillance for TB.^60,61^

### Strengths and limitations

This study is a cross-sectional survey of TB room nurses who worked in close proximity to CHW teams. Their views of the community-based TB screening services were considered objective enough to understand the prevailing strengths and limitations of these services in their context. The results can only be seen as the views of these personnel and may not be entirely accurate in instances where the TB room nurse was new or had limited encounters with the CHWs and/or only worked with an NGO in providing targeted TB services within the locale. Also, respondents may have been biased in their level of agreement with statements that reflected on the quality of their own TB room services. This may be reason for the overly optimistic views regarding the coordination of services with NGOs and community-based services.

The survey could have included OTLs and CHWs but it would have been difficult to reach this group with a sufficient response rate with the resources available for the study. It is also important to highlight the limitations experienced with conducting a paperless survey during a pandemic, when HCWs had many other priorities. The response rate was however considered acceptable. It is not clear if the non-responders differed in any way from the respondents; when they did not respond, it was presumed to be due to non-consent for inclusion in the survey. The use of electronic questionnaires may have discouraged persons who were not comfortable with the use of such mediums of communication or were worried about using their own cellphone data. The failure to respond could also be considered a bias towards better-resourced clinics as they had better access to the use of electronic devices.

### Implications and recommendations for practice, further research

Managers need to ensure that WBPHCOTs have the resources they need to perform effectively, in particular, sufficient numbers of dedicated OTLs and transport. As multiple programmes utilise the WBPHCOTs, it may be possible to source resources from better-funded programmes. The OTLs should ensure good relationships, coordination and support from the facility-based members of the primary health care PHC team. In addition, the WBPHCOTs should have the correct number of CHWs to cover the population adequately.

Managers of the TB programme should look at ways to build collaboration between community-based health and social services, particularly the Department of Social Development and South African Social Security Agency (SASSA). The coordination of goals and objectives at top levels of management would ultimately influence coordination of resources in the community. Better stakeholder engagement and multisectoral collaboration are needed.

The service agreements with NGOs should ensure that additional resources are allocated equitably to cover all facilities and communities. In addition, where it is necessary to supplement WBPHCOTs, they should provide active TB surveillance, rather than more focused activities.

The private sector should be engaged further as local GPs can assist with facility-based screening for TB.

The reach, acceptance, safety and effectiveness of CHWs in some communities can be strengthened through more active community engagement and participation. This needs to be driven by facility and subdistrict level managers. Community clinic committees and health forums need to be actively engaged. Community education and awareness could also be enhanced by digital technology and multimedia platforms.

There is a need to look at a model of care that sees the CHW as someone who facilitates wellness in the community beyond just the delivery of TB screening services, and a conduit for health and social service interventions. This will require further training of the CHWs to facilitate wellness consciousness in communities surrounding TB and other diseases.

Further research is required into how much can be accomplished with implementing active surveillance for TB in the Eastern Cape. It is also important to study how empowering communities to be active in improving their own health outcomes would facilitate wellness in communities. A quality improvement study is already ongoing as a follow-up to this study to try and put some of these recommendations into practice in one subdistrict. Table 9 provides an overview of the implications of the findings of this study and recommendations for addressing the challenges identified.

**TABLE 9:** Implications and recommendations.

Implication of findings	Recommendations	Personnel responsible for change
Inadequate make-up in teams	Increase the number of teams for adequate coverage. Increase the number of CHWs and OTLs available to serve on the teams.	District and subdistrict coordinators of WBPHCOT
The OTL should manage relationships with the facility-based healthcare workers	The OTL is obliged to engage with the facility-based HCWs for their continued support of the CHW teams to help build their capacity through the MDT.	Facility manager, OTL, CHW, district and subdistrict coordinators of WBPHCOT
There is a need to reorganise teams to realign with scarce human resources	Consider improving capacity of more experienced CHWs to lead teams under the guidance of professional nurses who manage several teams.	Policymakers, human resource managers, district and subdistrict coordinators of the community-based services
CHWs as generalists should ideally use a COPC approach to conduct their community-based services	A paradigm shift required with managers having a community-orientated approach to providing community-based services. Managers can source funding from some of the programmes in PHC to provide resources in community-based services, e.g. Maternal, Newborn, Child Health and Nutrition programme, etc. This is especially because the CHWs provide services relevant to all these. CHWs need to be capacitated to facilitate wellness in communities in addition to preventive services and minor curative services including linkage to care.	Policymakers, district, subdistrict and facility managers
Coordination of CHW team resources equitably in the community-based services	Service level agreements between the district health services and social partners should direct resources to the areas of need in the community-based services.	Top managers in the district health services, district and subdistrict coordinators of community-based services.
Coordination of services between community-based services and social support services	Better stakeholder engagement between multiple stakeholders providing services in the community to provide targeted interventions that address social determinants of health known to foster transmission of communicable diseases in communities. Clear pathways for referral between community-based services and the department of social development and SASSA.	Managerial structures within the district and subdistrict drive the multisectoral engagement. CHWs, OTLs, professional nurses refer clients as necessary.
Community engagement	Clear pathways for community engagement should be facilitated through the clinic community health forums. OTLs and CHW teams should engage with identified community leaders to facilitate opportunities for community engagement and community participation initiatives.	Facility management, subdistrict coordinator of the community-based services, OTLs, CHWs
Further research	Further research in primary health care that evaluates the implementation of the policy on the provision of community-based services by CHWs. Further research including community surveys on the effect of the community-based services.	Researchers, clinician scientists

CHW, community health worker; TB, tuberculosis; PHC, primary health care; OTL, outreach team leader; WBPHCOT, ward-based primary health care outreach teams; SASSA, South African Social Security Agency; COPC, community oriented primary care; MDT, multidisciplinary team.

## Conclusion

Even though the National Department of Health (NDoH) has adopted active surveillance for TB as a policy to combat the TB epidemic, it is only available from WBPHCOTs in 67.3% of catchment areas served by these clinics. Clinics mostly relied on contact tracing and screening of patients at the facilities. The implementation of community-based active surveillance by CHWs was limited by several factors: a lack of team leadership, a lack of transport, security concerns and lack of time to focus on TB screening. It was shown that respondents believed that inaccurate home addresses and contact details, poor collaboration with social services and limited affiliation of patients to specific clinics influenced outcome of TB screening services. There were some differences between rural and urban districts suggesting the context-specific nature of challenges in both districts. It is recommended that resources, facility support and team leadership for CHW teams be improved. Community and stakeholder engagement is essential to ensure coverage, safety, acceptance and effectiveness of active TB surveillance.
